# The Global Leprosy Assessment Index (GLAI): A new approach for measuring the severity of disease in Brazil

**DOI:** 10.1016/j.imj.2023.04.008

**Published:** 2023-05-03

**Authors:** Lucas Silva, Thiago Rocha, Dalson Figueiredo Filho

**Affiliations:** a Department of Medicine, Universidade Estadual de Ciências da Saúde do Estado de Alagoas, Maceio 57010-382, AL, Brazil; b Department of Political Science, Universidade Federal de Pernambuco, Recife 50670-901, PE, Brazil

**Keywords:** Leprosy, Transmissible disease control, Public health policy, Health indicator, Factor analysis

## Abstract

**Background:**

In Brazil, the Ministry of Health (MH) monitors leprosy using 15 indicators, with the aim of implementing and evaluating evidence-based public policies. However, an excessive number of variables can complicate the definition of objectives and verification of epidemiological goals.

**Methods:**

In this paper, we develop the Global Leprosy Assessment Index (GLAI), a composite measure that integrates two key dimensions for the control the disease: epidemiological and operational. Using a confirmatory factor analysis model to examine 2020 state-level data, we have standardized GLAI to a range of 0 to 1.

**Results:**

Higher values within this range indicate a greater severity of the disease. The mean value of the GLAI was 0.67, with a standard deviation of 0.22. Roraima has the highest value, followed by Paraíba with 0.88 while Tocantins records the lowest value of the indicator, followed by Mato Grosso with 0.14. The epidemiological and operational indicators have a positive but statistically insignificant correlation (*r* = 0.25; *p*-value = 0.20).

**Conclusions:**

The development of evidence-based public policies depends on the availability of valid and reliable indicators. The GLAI presented in this paper is easily reproducible and can be used to monitor the disease with disaggregated information. Furthermore, the GLAI has the potential to serve as a more robust parameter for evaluating the impact of actions designed to eradicate leprosy in Brazil.

## Graphical abstract

**Figure:**
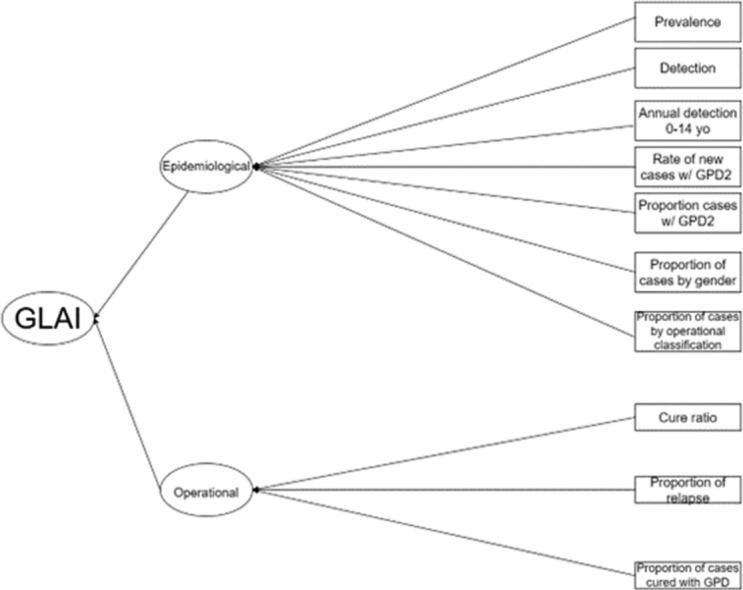

## 1 Introduction

Leprosy, also known as Hansen's disease, is a chronic infection caused by *Mycobacterium leprae*. The disease can lead to various types of neurological damage in those who are infected [1]. In Brazil, the standard treatment protocol for leprosy involves a single polychemotherapy regimen (PQTU in Portuguese) that includes the use of clofazimine, dapsone, and rifampicin [2]. The nationwide adoption of PQTU has effectively decreased the prevalence of leprosy in the country [3].

Despite advances in treatment, leprosy continues to be a major public health concern [3]. According to the World Health Organization (WHO), Brazil has the second highest number of new cases in the world, with only India reporting more [4]. From 2015 to 2019, 137,385 new cases of the disease were reported in the country [2] with cases reported in various regions of the country. The North and Midwest regions had the highest proportions of reported cases, at 37.3% and 35.2%, respectively [2].

The Ministry of Health (MH) has implemented several institutional policies aimed at reducing the prevalence of leprosy [5]. These policies mainly focus on early diagnosis and treatment of cases, which requires strengthening primary care services [6]. In the Guidelines for Surveillance, Care, and Elimination (2016), the MH has defined 15 indicators aimed at monitoring progress towards elimination (epidemiological) and assessing the quality of leprosy services (operational) [7].

Indicators play a crucial role in implementing and evaluating government programs [8]. However, the presence of numerous parameters can make it challenging to define objectives and monitor established goals. This can ultimately hinder the effectiveness of public policies and the assessment of progress in combating the problem [9].

This article introduces the Global Leprosy Assessment Index (GLAI), a composite measure that provides a single, transparent, and reproducible procedure to assess the severity of the disease in Brazil.

## 2 Materials and methods

### 2.1 Data and variables

The epidemiological and operational monitoring indicators defined in the Brazilian Ministry of Health's Guidelines for Surveillance, Care, and Elimination of Leprosy are both summarized in Table 1.

**Table 1:** Indicators for tracking the progress of eradicating Leprosy as a public health concern.

Type	Name	Purpose
Monitoring the progress of elimination of leprosy (epidemiological)	Annual prevalence rate of leprosy per 10,000 inhabitants	Measure the magnitude of the endemic
	Annual detection rate of new cases of leprosy per 100,000 inhabitants	Measure morbidity strength, magnitude and trend of the endemic
	Annual detection rate of new cases of leprosy in the population aged 0–14, per 100,000 inhabitants	Measure the strength of recent transmission of the endemic and its trend
	Rate of new cases of leprosy with grade 2 of physical disability (GPD2) at the time of diagnosis per 100,000 inhabitants	Evaluate the deformities caused by leprosy in the general population and compare them with other disabling diseases. Used in conjunction with the detection rate for monitoring the trend of early detection of new cases of leprosy
	Proportion of cases of leprosy with grade 2 of physical disability (GPD2) at the time of diagnosis among new cases detected and evaluated in the year	Evaluate the effectiveness of early detection and/or early detection activities
	Proportion of cases of leprosy cured with grade 2 of physical disability (GPD2) among cases evaluated at the time of discharge for cure in the year	Evaluate the transcendence of the disease and support the planning of post-discharge prevention and treatment activities for disabilities
	Proportion of cases of leprosy, by gender among new cases	Evaluate the capacity of services to assist leprosy cases
	Proportion of cases by operational classification among new cases	Evaluate cases at risk of developing complications and for proper replenishment of PQTU
	Rate of detection of new cases, by race/color among population of respective races/colors	Measure the magnitude of the endemic by race/color

Evaluate the quality of leprosy services (operational)	Proportion of leprosy cure among cases diagnosed in the years of the cohorts	Evaluate the quality of care and follow-up of newly diagnosed cases until completion of treatment
	Proportion of cases of leprosy in treatment abandonment among cases diagnosed in the years of the cohorts	Evaluate the quality of care and follow-up of newly diagnosed cases until completion of treatment
	Proportion of contacts examined of new cases of leprosy diagnosed in the years of the cohorts	Measures the ability of services to conduct surveillance of contacts of new cases of leprosy, increasing the detection of new cases
	Proportion of relapse cases among the cases reported in the year	Identifying municipalities that report relapse cases for monitoring therapeutic failure
	Proportion of new cases of leprosy with assessed physical disability at the time of diagnosis	Measuring the quality of care in health services
	Proportion of cured cases in the year with assessed physical disability among new cases of leprosy in the cohort period	Measuring the quality of care in health services

*Source:* Adapted from Brasil [7].

Although comprehensive, these guidelines have yet to be effectively implemented by the agencies responsible for gathering and organizing epidemiological data. For instance, a significant number of these indicators are not present in the Health Information System (TABNET, in Portuguese) of the Department of Informatics of the Unified Health System (DATASUS, in Portuguese) [10]. Recently, the MH established a panel with indicators and basic data on leprosy in Brazilian municipalities [11]. However, this platform does not include important information necessary for effective monitoring of the disease, such as the percentage of cases that have been cured but still have physical disability and the percentage of patients who have discontinued treatment.

The Department of Chronic Conditions and Sexually Transmitted Diseases (DDCCIST, in Portuguese) of the MH publishes information on the epidemiological status of various diseases, including leprosy [12]. Although the variables measured do not include all of the items listed in Table 1, it is the closest source to the proposed model. Due to these issues related to data availability, GLAI is composed of a limited number of variables, as shown in Table 2.

**Table 2:** Explanation of the variables utilized in constructing the Global Leprosy Assessment Index (GLAI).

Dimension	Indicator
Epidemiological	Annual rate of leprosy prevalence per 10,000 inhabitants
	Annual detection rate of new cases of leprosy per 100,000 inhabitants
	Annual detection rate of new cases of leprosy in the population aged 0–14, per 100,000 inhabitants
	Rate of new cases of leprosy with grade 2 of physical disability at the time of diagnosis per 100,000 inhabitants
	Proportion of cases of leprosy with grade 2 of physical disability at the time of diagnosis among new cases detected and evaluated in the year
	Proportion of cases of leprosy, by gender among new cases
	Proportion of cases by operational classification among new cases

Operational	Proportion of leprosy cure among cases diagnosed in the years of the cohorts
	Proportion of relapse cases among cases reported in the year
	Proportion of cured cases in the year with physical disability evaluated among new cases of leprosy in the cohort period

This research employs a state-level analysis, incorporating data from 2020, the most current period of availability, with the 26 states and Federal District serving as the unit of analysis.

### 2.2 Statistical analysis

Statistically, factor analysis technique will be used, which enables reducing a large number of observed variables to a smaller set of factors/dimensions [13]. This method is a key tool for measuring constructs [14].

**Fig. 1:**
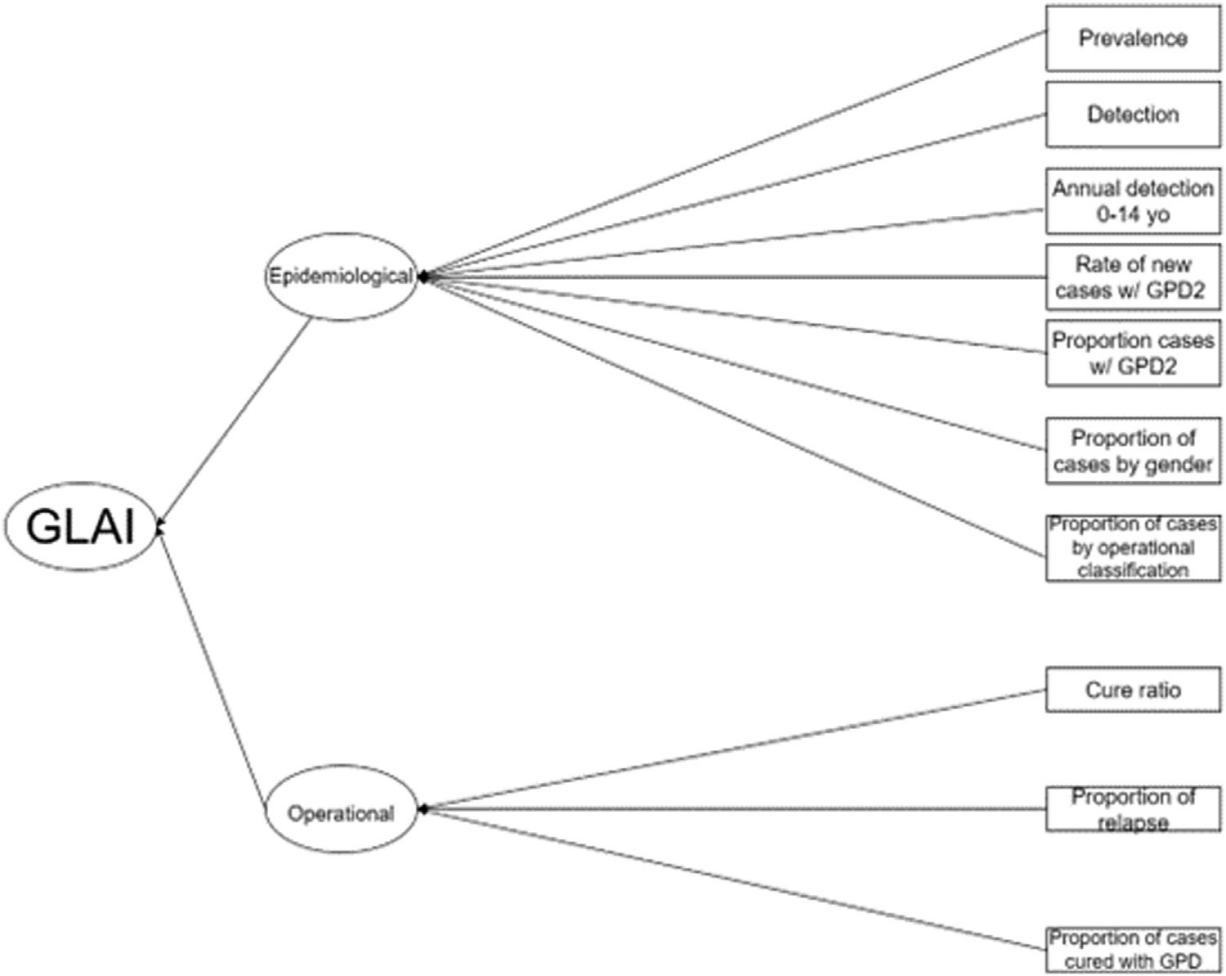
Global Leprosy Assessment Index (GLAI) dimensions and indicators.

Factor analysis can be approached from two perspectives: (1) exploratory and (2) confirmatory. The former is typically employed in earlier stages of research to explore the data [13] whereas the latter is utilized to test hypotheses, and the researcher is guided by more clearly defined theoretical expectations [15]. In this study, we employ a confirmatory perspective with the aim of evaluating the extent to which the indicators recommended by the MH can be used to construct a synthetic measure of leprosy assessment in Brazil. Figure 1 illustrates the conceptual model for the representation of the GLAI.

The final outcome of factor analysis is standardized scores with a mean of 0 and standard deviation of 1. Initially, the models will be run separately for each dimension, and the scores will be normalized, modularly, between 0 and 1 to make the index easier to understand.

The interpretation of the values varies depending on each dimension. For the epidemiological dimension, values closer to 1 indicate a more severe situation in that location, whereas values closer to 0 indicate a better situation with a lower incidence of cases. For the operational dimension, values closer to 1 indicate more effective health services in combating the disease, whereas values closer to 0 indicate a poorer evaluation of the activities.

After that, a new model will be run to construct the GLAI from the previously obtained standardized scores for each dimension. According to best practices in literature, a weighted average of the standardized values will be calculated using the accumulated variance percentage of each dimension in the composition of the index, to ensure that each dimension contributes proportionately to the final estimate. Equation (1) shows the algebraic definition of the GLAI

(1) $$\text{GLAI}=z_{epidemiological}\sigma_{epidemiological}^{2}\;+\;z_{operational}\sigma_{operational}^{2}$$

Where *z* indicates the standardized score extracted individually from each dimension and *σ*² indicates the accumulated variance of each corresponding dimension in the GLAI. As with each dimension, the value of the GLAI will be normalized between 0 and 1 to facilitate understanding of the variable. The closer to 1, the better the situation of the location, both epidemiologically and operationally.

### 2.3 Computational tools

All statistical analyses were performed using *R Statistics* software, version 4.0.5. *Microsoft Excel 2020* was used to tabulate and standardize the data in an editable spreadsheet format, as the information was originally provided in a portable document format (.pdf). Materials for replication, including databases and computational scripts, are available in public repositories such as the *Open Science Framework (OSF)*.

## 3 Results

**Fig. 2:**
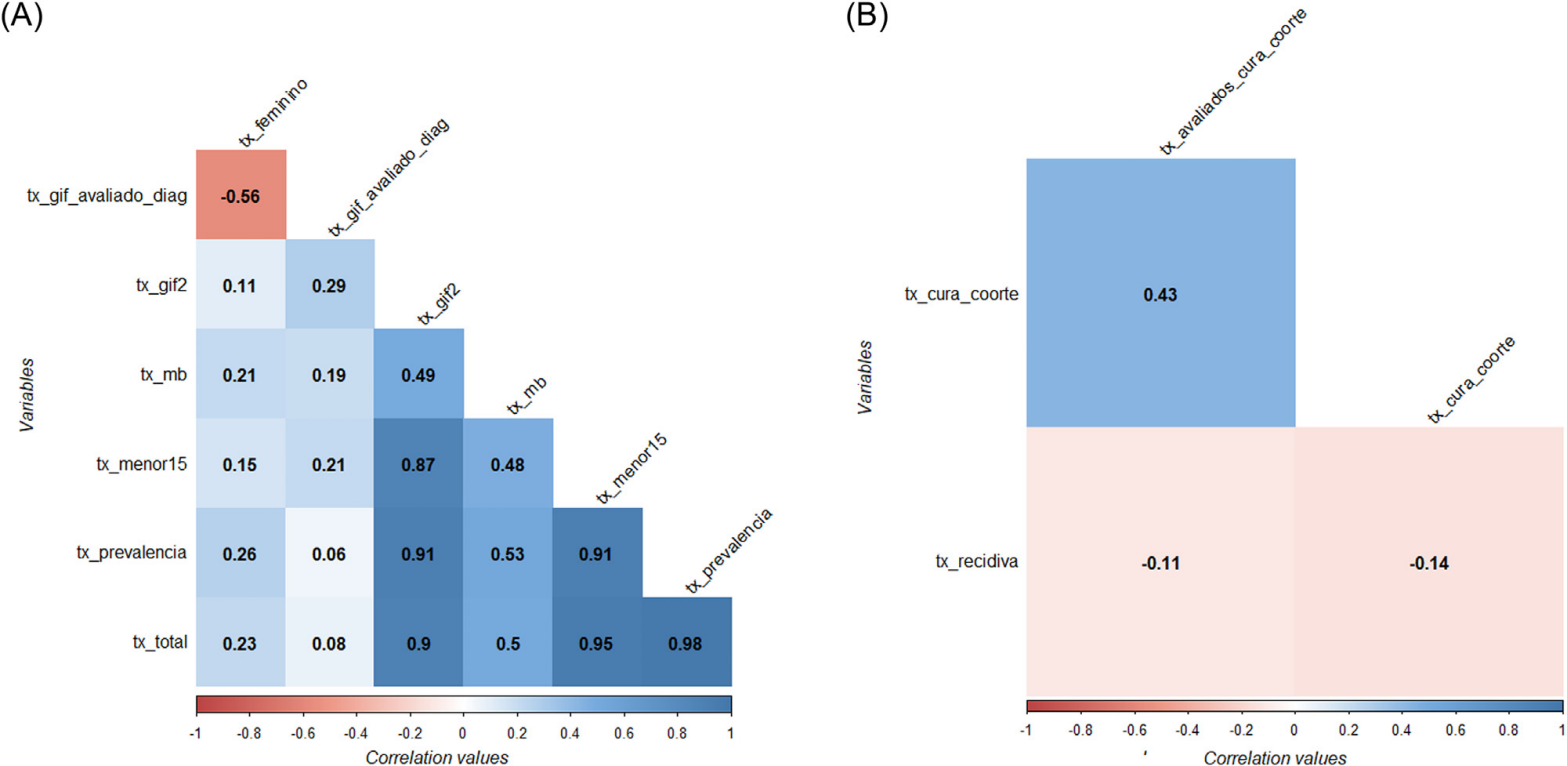
Correlation pattern between the analyzed variables. (**A**) describes the correlation pattern of the epidemiological dimension, (**B**) exposes that of the operational dimension.

The first step in a factor analysis model is to analyze the degree of correlation between variables. Hair et al. [16] point out that the correlation matrix should show values above 0.3. Figure 2 shows the pattern of linear association between variables.

Figure 2A illustrates the correlation pattern between the variables of the epidemiological dimension. Of the 21 associations, 11 meet the assumption suggested by Hair et al. [16] in terms of magnitude and statistical significance. For the operational dimension, represented in Figure 2B, only one correlation meets the criterion.

The next step is to analyze the sample's suitability through the Kaiser-Meyer-Olkin (KMO) and Bartlett's sphericity (BTS) tests [13]. The KMO is an indicator that varies from 0 to 1. The closer to 1, the better. In literature, there are different ways to categorize this value. However, the acceptable limit for performing factor analysis is that it is greater than 0.5 [16]. In turn, for BTS, the literature recommendation is that values lower than 0.05 indicate an acceptable solution [13]. Hair et al. [16] argue that variables should present communalities patterns above 0.4. Table 3 shows the sample suitability test statistics for each dimension.

**Table 3:** Statistics of factor analysis models for each dimension.

Parameters	Dimension	
	Epidemiological	Operational
KMO	0.70	0.53

BTS	207.98 (*p-*value < 0.001)	5.69 (*p-*value = 0.12)

Commonalities	tx_prevalencia = 0.74 tx_total = 0.68 tx_menor15 = 0.73 tx_gif2 = 0.84 tx_gif_avaliado_diag = 0.32 tx_mb = 0.7	tx_cura_coorte = 0.52 tx_recidiva = 0.69 tx_avaliados_cura_coorte = 0.52

Explained variance	70%	81%

In terms of the epidemiological dimension, both KMO and BTS fall within the parameters established by literature. However, the variable tx_gif_avaliado_diag presents a community lower than 0.4, which means, in principle, that it should be excluded from the measurement model. However, due to the fact that the model was developed in a confirmatory perspective, it was decided to keep this variable once it theoretically corresponds to the examined construct. In total, the variables that represent this dimension explain about 70% of the variance.

For the operational dimension, all variables have commonalities above 0.4. However, KMO and BTS are not in line with what is established, which suggests that these variables do not exhibit a well-defined pattern of linear association. The 3 variables that make up this dimension explain approximately 81% of the construct.

**Fig. 3:**
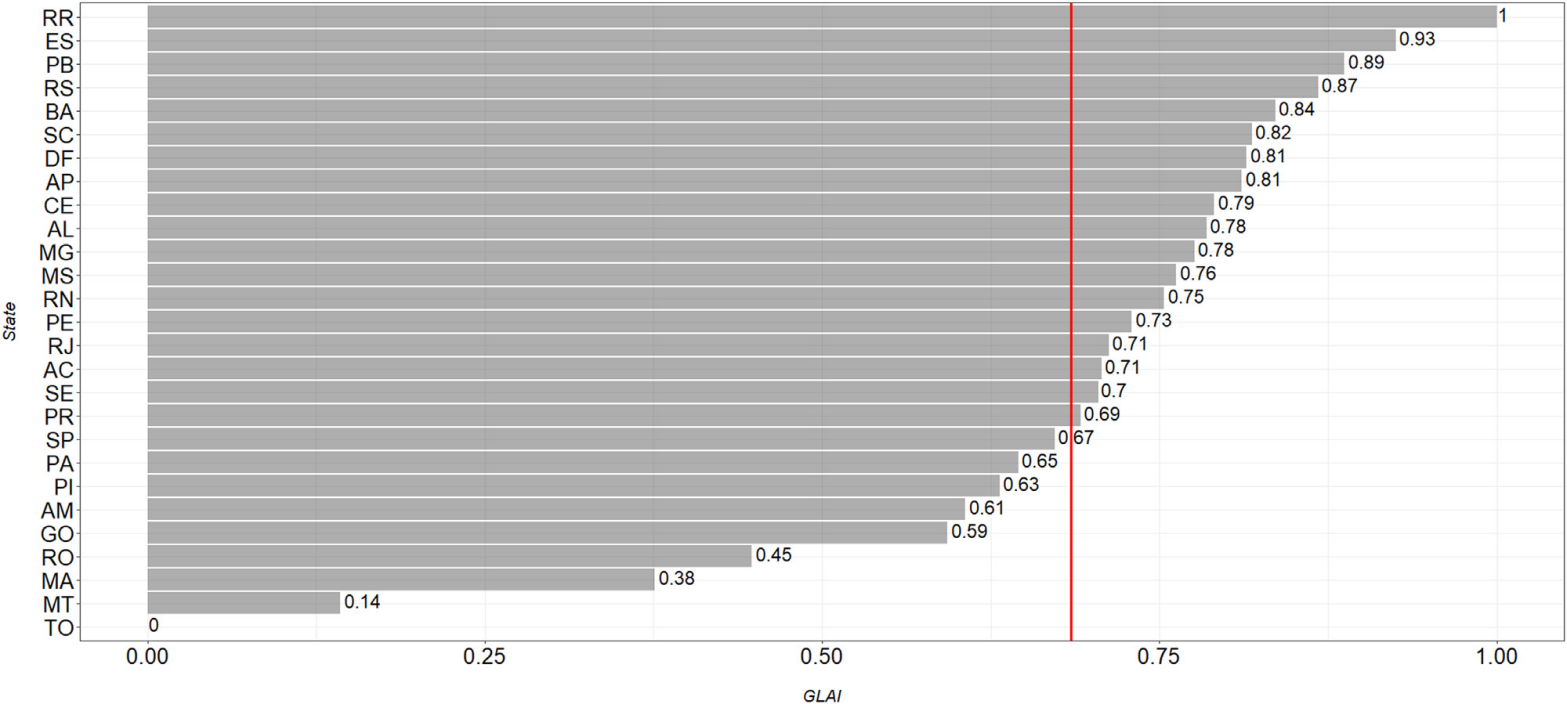
GLAI distribution pattern among Brazilian states (2020). Note: AC, Acre; AL, Alagoas; AP, Amapá; AM, Amazonas; BA, Bahia; CE, Ceará; DF, Distrito Federal; ES, Espírito Santo; GO, Goiás; MA, Maranhão; MT, Mato Grosso; MS, Mato Grosso do Sul; MG, Minas Gerais; PA, Pará; PB, Paraíba; PR, Paraná; PE, Pernambuco; PI, Piauí; RJ, Rio de Janeiro; RN, Rio Grande do Norte; RS, Rio Grande do Sul; RO, Rondônia; RR, Roraima; SC, Santa Catarina; SP, São Paulo; SE, Sergipe; TO, Tocantins.

Figure 3 displays the distribution of the GLAI by federal unit. Roraima has the highest value (1), followed by Paraíba (0.88) and Rio Grande do Sul and Espírito Santo (0.86). In other words, these states have better evaluation scores in the fight against the disease. On the other hand, Tocantins records the lowest value of the indicator, followed by Mato Grosso (0.14) and Maranhão (0.37), which represents a weakening of the eradication actions of the disease in these states. The red line indicates the average of the GLAI recorded in the country (0.67), which has a standard deviation of 0.22.

**Fig. 4:**
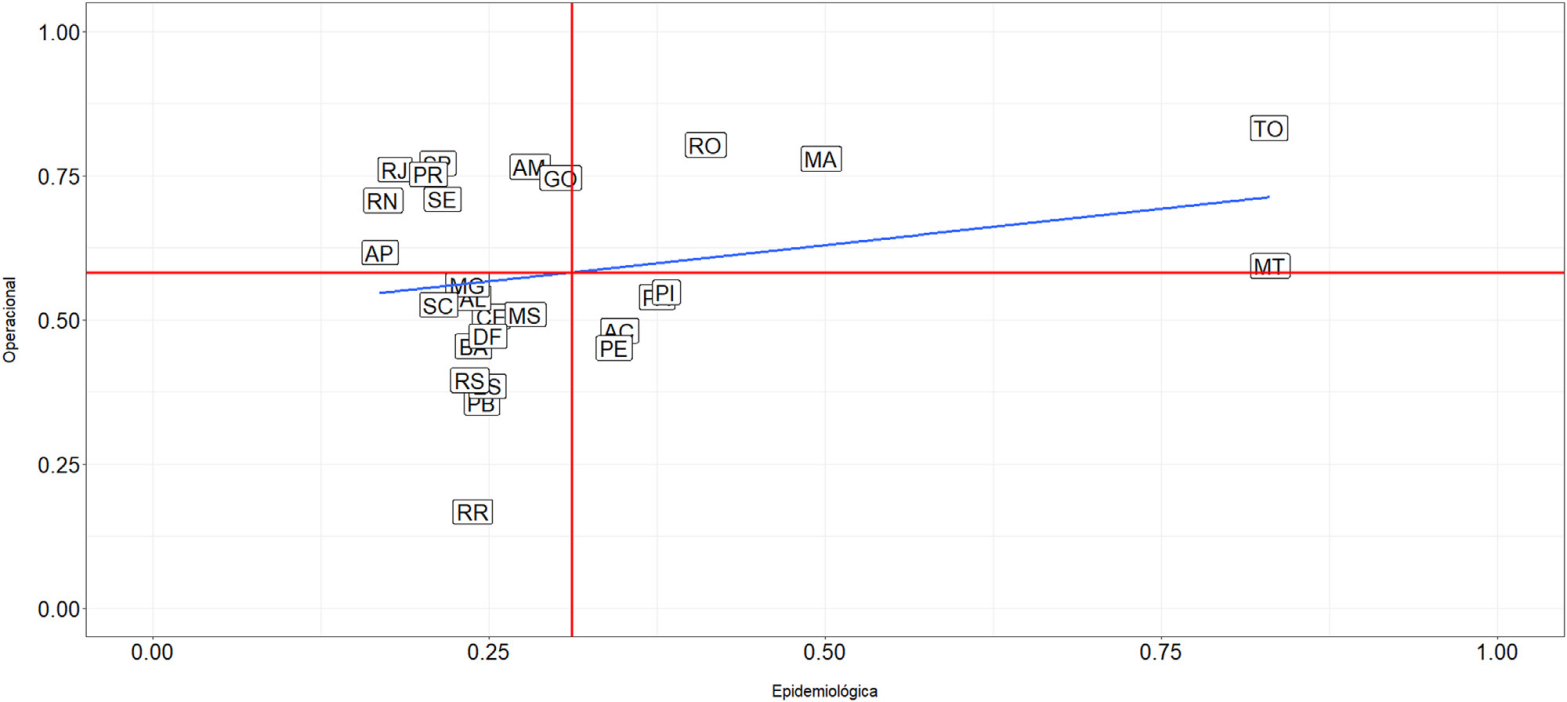
Dispersion pattern between the epidemiological and operational dimensions of the GLAI. *Note*: AC, Acre; AL, Alagoas; AP, Amapá; AM, Amazonas; BA, Bahia; CE, Ceará; DF, Distrito Federal; ES, Espírito Santo; GO, Goiás; MA, Maranhão; MT, Mato Grosso; MS, Mato Grosso do Sul; MG, Minas Gerais; PA, Pará; PB, Paraíba; PR, Paraná; PE, Pernambuco; PI, Piauí; RJ, Rio de Janeiro; RN, Rio Grande do Norte; RS, Rio Grande do Sul; RO, Rondônia; RR, Roraima; SC, Santa Catarina; SP, São Paulo; SE, Sergipe; TO, Tocantins.

Figure 4 illustrates the pattern of dispersion between the dimensions. The red vertical line represents the average of the epidemiological dimension (0.22), while the red horizontal line represents the average of the operational dimension (0.62). The blue line represents the linear fit line between the latent variables, which has a correlation coefficient of 0.25 (*p-value* = 0.20).

To ease interpretation, the graph is divided into quadrants based on the averages of each axis. States located in Quadrant 1 (Rondônia, Maranhão, Tocantins, and Mato Grosso) have high disease incidence but good operational response. The worst scenario is found in Quadrant 2 (Pernambuco, Acre, Pará, and Piauí) where not only the presence of the disease is higher, but the states also do not have a satisfactory institutional response. Quadrant 3 has the largest number of states (Minas Gerais, Santa Catarina, Alagoas, Ceará, Mato Grosso do Sul, Distrito Federal, Bahia, Rio Grande do Sul, Espírito Santo, Paraíba, and Roraima) which despite not having high administrative action, these locations have lower incidence. The best assessment scenario is found in Quadrant 4, where the states of Rio de Janeiro, Paraná, São Paulo, Sergipe, Amazonas, Goiás, Amapá, and Rio Grande do Norte are located. These areas are characterized by low incidence of cases and better institutional response in combating the disease. However, it is important to note that the lack of variables related to the abandonment process may affect the precision of these findings, as this factor is directly linked to the quality of health services provided in combating the disease.

## 4 Discussion

As Table 1 shows, the diversity of parameters for monitoring leprosy in the country has resulted in various studies in literature with different analytical methods. Generally, these studies tend to concentrate on a specific set of indicators rather than a more comprehensive assessment.

At the national level, Souza et al. [17] evaluate the trend of epidemiological and operational indicators of leprosy in the country between 2001 and 2017. The results indicate a decreasing trend in the overall number of cases, those under 15 years old, prevalence, and GPD2/1 million inhabitants.

Ribeiro et al. [18] evaluate the indicators in the country between 2005 and 2015. They observe that the prevalence of the disease was at a moderate level, with a decreasing national trend despite regional variations. There was a reduction in the coefficient of new cases in individuals <15 years old, as well as a decrease in the diagnosis of new cases with a GPD2. The cure rate remained regular.

At the subnational level, the number of studies is greater and more diverse. Pereira et al. [19] observe the indicators of leprosy in Teresina (state of Piauí) between 2001 and 2008 and detect a hyperendemic character in the city, which can lead productive-age people to become inactive.

In Paraná, Oliveira et al. [20] observe the variation of indicators in the municipalities of Curitiba, Londrina, and Foz do Iguaçu between 2001 and 2010. Despite the findings pointing to a reduction in diagnoses, the authors warn of the existence of hidden cases in these cities. On the other hand, they also detect a slight improvement in services provided to the population.

Imbiriba et al. [21] examine the data from Manaus (state of Amazonas) between 1998 and 2005 and point out that cases of leprosy in those under 15 years old accounted for 10.4% of the total. They argue that the coefficient was at a hyperendemic level between 1998 and 2003 and suffered a small reduction from 2004.

On the other hand, in Amapá, between 2005 and 2018, Basso, Andrade, and Silva [22] detected a decreasing trend in new cases and the rate in those less than 15 years old, with fluctuations in the rate of new cases with disabilities. In the authors' view, a hidden endemic scenario is found, with active transmission and late diagnosis.

Similar conclusions were obtained by Monteiro et al. [23] when analyzing the cases of the disease in the state of Tocantins (2001–2012). There was a significant and decreasing trend for the overall detection and proportion of paucibacillary cases, stability in cases <15 years old, and detection of cases with GPD2. Lima et al. [24] also observed a reduction in detection, while the proportions of GPD2 and examined physical disability increased in the state of Goiás between 2001 and 2017.

In Maranhão, Ancheita et al. [25] observed 77,697 cases of leprosy in the general population and 7,599 in those under 15 years old between 2011 and 2015. In general, a decreasing trend in the coefficients of the populations analyzed was detected. In addition, a stationary trend was observed in the proportion of leprosy cures.

Souza et al. [26] also observe poor performance in operational indicators in Bahia between 2001 and 2014. The evidence indicates that most municipalities in the state have a cure percentage lower than 75%. Additionally, about 55.3% of Bahian municipalities had high occurrence of GPD2 at the time of diagnosis.

In addition to spatial and temporal differences, another factor that differentiates these studies is the amount and types of indicators used in obtaining the findings. From the perspective of public policies, these variables are relevant tools in monitoring the effectiveness of policies in achieving their objectives [27]. They help to understand whether the actions are in line with the objectives [27].

However, the presence of multiple indicators can interfere in the process of policy evaluation [28]. If the lack of information makes it difficult to accurately examine the actions, the excess also causes the same problem, since managers and researchers can be hampered in the decision-making process of which information to use and how to analyze it [29,30].

The number of indicators defined by the MH for evaluation is noteworthy. None of the studies present in the literature or even the epidemiological bulletins produced by the health authority itself, which are released annually, analyze all variables together. It is common to use proxies, which is the selection of one or another variable that represents a dimension, whether operational or epidemiological. In the end, this can make the process of monitoring and evaluating the policy more difficult [31].

The lack of indicators from the guidelines in official data repositories is one of the chronic problems related to information systems in the country [32]. During the data collection process, some information of interest was not present in either DATASUS or the panel. This situation, in addition to hindering the quality of the analysis produced around the disease [32], can lead to a redefinition of the parameters for evaluating the policy. The quality of the data is also a factor that affects the reliability and validity of the measures. Furthermore, the sample used was small, and the number of variables for the construction of the indicator was limited [16]. Finally, the confirmatory model is subject to the occurrence of theoretical errors, as some theoretically proposed items may not adequately measure the meaning of the construct [33].

The process of constructing composite indicators, such as the GLAI, serves to aggregate a series of observed variables, based on an underlying model of the concept being measured [34]. Indices such as GLAI can provide more comprehensive performance evaluations and present an overall picture in a way that is accessible to a variety of audiences [34]. Additionally, it can facilitate the process of setting goals and obtaining results [9].

However, it is crucial to note that aggregate constructs leave out individual variables that may be crucial in evaluating results at a more detailed level. For this reason, researchers should carefully consider the type of data they have and how they wish to evaluate the results of the policy.

## 5 Conclusions

This study advances the literature on the topic by developing a useful, transparent, and reproducible index to evaluate the severity of Hansen's disease in Brazil. This development is anticipated to facilitate the formulation and execution of targeted public policies to eliminate the disease within the country.

## Funding

Coordenação de Aperfeiçoamento de Pessoal de Nível Superior (CAPES), Conselho Nacional de Desenvolvimento Científico e Tecnológico (CNPQ), and Fundação de Amparo à Pesquisa do Estado de Alagoas (FAPEAL).

## Author contributions

L.S.: Study conception and design; data interpretation and statistical analysis; manuscript writing; T.M.: Study conception and design; revision of the manuscript; D.F.F.: Statistical analysis; writing and revision of the manuscript.

## Declaration of competing interests

The authors declare that they have no known competing financial interests or personal relationships that could have appeared to influence the work reported in this paper.

## Data available statement

Replication materials, including raw data and computational scripts, are available on <https://osf.io/zjeyu/>.

## Ethics statement

Not applicable – retrospective study of secondary and public data.

## Informed consent

Not applicable.
